# Return to work after cell transplantation in patients with angiitis-induced critical limb ischaemia and factors related: a single-centre retrospective cohort study

**DOI:** 10.1186/s13287-022-02807-1

**Published:** 2022-04-01

**Authors:** Hao Liu, Yifan Liu, Tianyue Pan, Yuan Fang, Gang Fang, Xiaolang Jiang, Bin Chen, Zheng Wei, Shiyang Gu, Peng Liu, Weiguo Fu, Zhihui Dong

**Affiliations:** 1 Department of Vascular Surgery of Zhongshan Hospital, Fudan University, 180 Fenglin Road, Shanghai, 200032 China; 2 Department of Hematology of Zhongshan Hospital, Fudan University, 180 Fenglin Road, Shanghai, China

**Keywords:** Critical limb ischaemia, Cell transplantation, CD34^+^, Return to work

## Abstract

**Background:**

Angiitis-induced critical limb ischaemia (AICLI) patients, who are usually young and have a high amputation rate, always lose their ability to return to the labour force. Return to work (RTW) not only indicates patients’ physical health, showing that they could undertake the work, but also demonstrates their psychological well-being. While cell transplantation showed satisfactory efficacy in limb salvage, few studies of AICLI patients’ RTW after transplantation have been reported.

**Methods:**

From May 2009 to May 2021, AICLI patients who underwent cell transplantation and completed no less than 12 months of follow-up were retrospectively enrolled. The primary endpoint was RTW. Patient demographics and characteristics of the ischaemic limbs were reviewed to analyse independent risk factors for RTW.

**Results:**

A total of 171 AICLI patients (170 males) were enrolled with a mean age of 41.9 ± 9.6 years (range: 20–57 years). The 12-month and 24-month RTW cumulative rates were 69.4% (95% confidence interval [CI] 61.6–75.6%) and 70.1% (95% CI 62.3–76.2%), respectively. Age < 40 years (odds ratio [OR] 2.659, 95% CI 1.138–6.719) and preoperative occupation as a mental worker (OR 8.930, 95% CI 2.665–42.847) were identified as independent protective factors for RTW. Perioperative limb infection with ulcer or gangrene (OR 0.250, 95% CI 0.075–0.779) was identified as an independent risk factor.

**Conclusion:**

AICLI patients who underwent cell transplantation usually had a satisfactory midterm RTW cumulative rate. AICLI patients < 40 years old with preoperative occupation as mental workers were more likely to return to work. Prevention of limb infection during the perioperative period is of great significance to RTW.

## Introduction

Angiitis-induced critical limb ischaemia (AICLI) patients, which are defined as patients with limb ischaemia caused by thromboangiitis obliterans (TAO) or other arteritis-related autoimmunological diseases such as systemic lupus erythematosus (SLE), psoriasis, and Crohn's disease, are usually relatively young and have a high amputation rate. Unfortunately, AICLI patients often have an impaired ability or an inability to return to the labour force due to intolerable rest pain, ulcers or even gangrene, thus posing a heavy burden to families and society [1]. Currently, cell therapy has shown satisfactory results in treating AICLI [2–5], and the ability to work after cell therapy is an important indicator of recovery for AICLI patients. Return to work (RTW) not only encompasses patients’ physical health status, indicating that they could undertake the work, but also reflects their psychological well-being [6, 7], and it decreases the financial burden on patients, their families and society [8]. On the other hand, the failure to return to work might increase patients’ incidence of depression and reduce their quality of life [9–11].

To the best of our knowledge, no previous studies of RTW after intervention for AICLI patients have been reported. As a site with a relatively large number of AICLI patients in China, our centre initialized cell therapy (peripheral blood mononuclear cells [PBMNCs] and purified CD34^+^ cells [PCCs] transplantation) for AICLI patients in 2009 and has accumulated valuable experience since then [2, 4, 5, 12]. To report the outcomes of AICLI patients’ RTW after cell therapy and to further identify the factors associated with a failure to RTW, we retrospectively reviewed and analysed the data of over 190 AICLI patients during the past 11 years.

## Materials and methods

### Patients and data collection

This study was approved by the Committee for the Protection of Human Subjects at Zhongshan Hospital, Fudan University. Written informed consent was obtained from each patient included in the study. It was performed in agreement with the ethical principles of the Declaration of Helsinki.

A consecutive cohort of patients from May 2009 to May 2021 was retrospectively enrolled in our centre and included 192 AICLI patients who received PBMNCs or PCCs transplantation. The inclusion and exclusion criteria for cell transplantation were described previously [4]. Briefly, the cohort included males and females aged between 18 and 80 years with AICLI (Rutherford classification [RC] 4–5), which was confirmed by clinical manifestation and computed tomographic angiography (CTA), magnetic resonance angiography (MRA), or digital subtraction angiography (DSA). The exclusion criteria were (1) serious health events (including but not limited to a myocardial infarction, cerebral apoplexy, pulmonary embolism, and severe hepatic and renal dysfunction) < 3 months before admission, (2) suspicion or diagnosis of malignancy at baseline, or (3) a life expectancy of no more than 6 months. In the current study, we also excluded patients who (1) already retired before the onset of AICLI or reached retirement age (≥ 60 years old for men and ≥ 55 years old for women) by the 12-month follow-up, (2) were lost to or died during the 12-month follow-up and (3) were transferred to another hospital due to unrelieved ischaemia or infection after discharge during the 12-month follow-up.

### Procedures for cell transplantation

As previously described [4], subcutaneous injections of rhG-CSF (Neupogen®; Amgen, Thousand Oaks, CA, USA) (5–10 μg/kg per day for 4 days) were employed to mobilize the bone marrow cells, and enoxaparin (4000 IU/day) was employed to prevent hypercoagulable states. On the fifth day, a suspension of PBMNCs was collected via leukapheresis (COM.TEC; Fresenius Hemocare GmbH, Bad Homburg, Germany). Then, after washing 3 times and resuspending the apheresis products in an ethylenediaminetetraacetic acid-phosphate buffered saline solution (200 mL) that contained 0.5% human albumin, the PBMNCs cell product was obtained. PCCs were obtained on the basis of PBMNCs by using a magnetic cell sorting system (Miltenyi-Biotec GmbH, BergischGladbach, Germany). The total cell count of CD34^+^ cells was determined by leukocyte counting and flow cytometry. The cell products were transplanted into the ischaemic limbs via equidistant intramuscular injections (0.5 mL/site) with patients under general anaesthesia.

### Data collection

During hospitalization, patients’ demographic characteristics, educational background, risk factors for cardio-and cerebrovascular diseases, disease history, treatment history, aetiology of AICLI, critical results of blood examination, and other data were recorded, and patients were asked about their employment status and their recent jobs before the onset of AICLI. The baseline features of the treated limbs were also recorded, including the number of involved limbs, the RC, the ankle-brachial index (ABI), the transcutaneous pressure of oxygen (TcPO_2_) of the dorsum, and the occlusion level of the arteries according to CTA, MRA, and/or DSA.

### Outcomes and follow-up

The primary endpoint was RTW, defined as part-time or full-time employment at 12 months after transplantation and further classified as return to the same job, switch to an easier job (relevant to AICLI) or normal job change (irrelevant to AICLI). The timepoint of RTW was also recorded. For patients who had not returned to work at 12 months after transplantation, reasons were classified as (1) unable to work owing to AICLI, (2) preferred not to work owing to AICLI (including full-time homemaker), (3) early retirement and (4) unable to or preferred not to work not directly owing to AICLI. Patients were required to pay regular clinical visits at 1, 3, 6, and 12 months and then annually after transplantation. Data related to the patients’ employment status were collected and recorded during the clinical visits or via telephone.

### Statistical analysis

The quantitative data, which were compared using Student’s t-test, are shown as the mean ± standard deviation (SD) or as the median with the interquartile range (IQR), depending on their distribution. Categorical variables, presented as frequencies and percentages, were compared using the χ^2^ test or Fisher’s exact test. Multivariable logistic regression was performed to identify the independent risk factors for RTW by a stepwise selection of variables. Factors with a *P* value less than 0.10 in univariate analyses were introduced into the multivariate model. The cumulative incidence of RTW was analysed by Kaplan–Meier analysis. All statistical tests were performed using a two-sided *α* of 0.05. All tests were performed using PASW software, version 19 (IBM Corporation, Armonk, NY, USA), or R, version 4.0.5.

## Results

### Baseline data

**Fig. 1:**
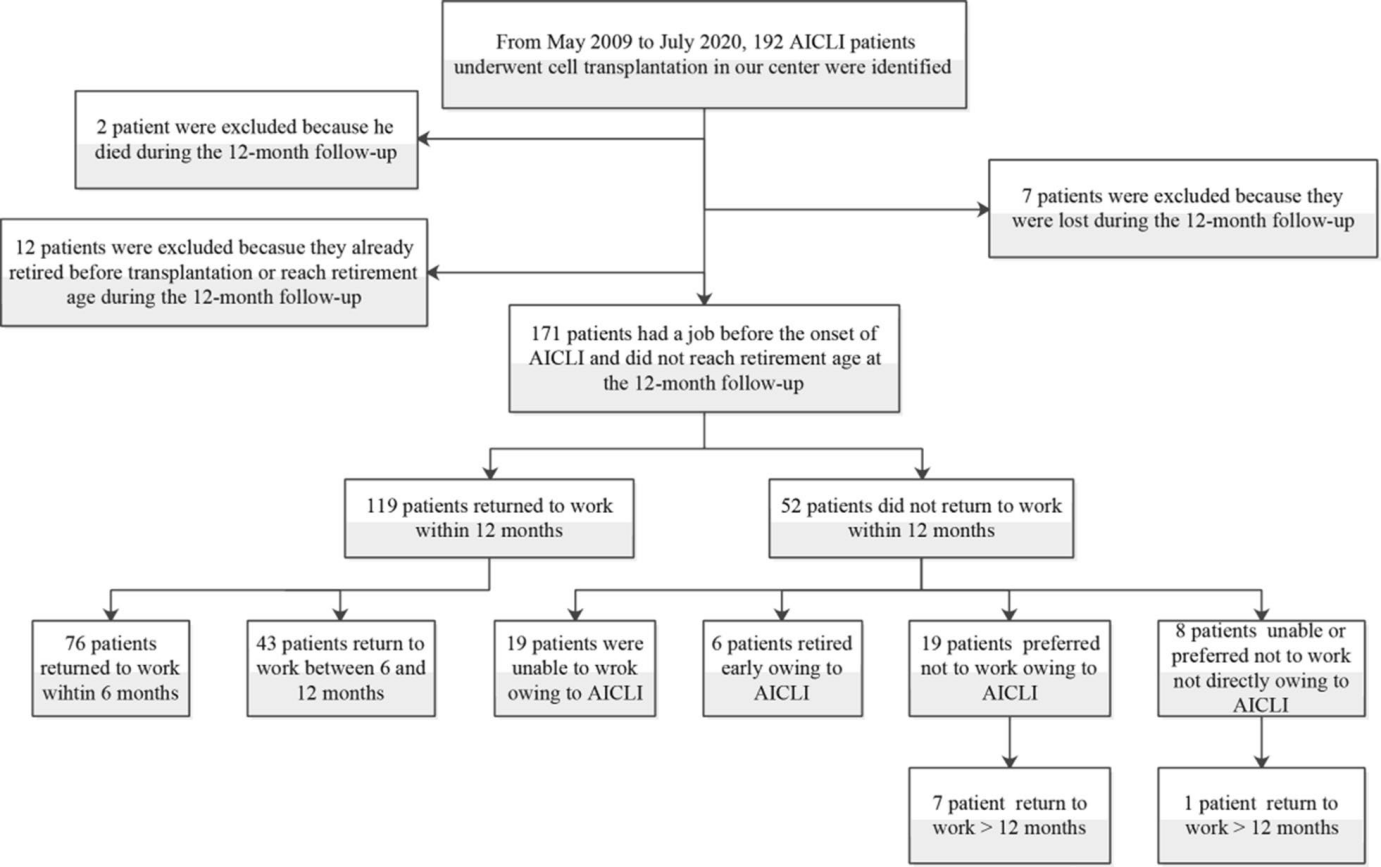
Protocol of current study. AICLI, angiitis-induced critical limb ischaemia

Between May 2009 and May 2021, 192 AICLI patients underwent cell transplantation in our centre. After excluding those who were lost to follow-up, retired before the onset of AICLI or reached retirement age during the 12-month follow-up, 171 patients with complete data at the 12-month follow-up were finally included and analysed (Fig. 1). The mean age of the patients was 41.9 ± 9.6 years (range 20–57 years), and the male ratio was 99.4% (170/171). The mean follow-up period was 57.9 ± 36.4 months (range 12–144 months). Among the 171 patients, 160 (93.6%) were married, 150 (87.7%) had children and 12 (7.0%) had received a college education. The patients were characterized by low frequencies of cardiovascular risk factors except for a high rate of smoking history (141 patients [82.5%]). All patients were in critical limb ischaemia (CLI) condition with RC 4 (22 patients [12.9%]) or 5 (149 patients [87.1%]), and most were TAO-induced (159 patients [93.0%]). There were 166 lower limbs and 13 upper limbs involved in the study. The median total CD34^+^ cell count in the transplants was 40.4 × 10^6^, with an IQR of 23.4–81.3 × 10^6^. More details of the baseline characteristics are shown in Table 1.

**Table 1:** Comparison of baseline characteristics between two groups of patients

	Total	RTW group	Non-RTW group	*P* value
	(*n* = 171)	(*n* = 119)	(*n* = 52)	
*Demographic characteristics*				
Age, years, mean ± SD	41.9 ± 9.6	40.9 ± 9.8	44.3 ± 8.9	0.033
20–29, *n* (%)	23 (13.4)	20 (16.8)	3 (5.8)	0.052
30–39, *n* (%)	39 (22.8)	29 (24.4)	10 (19.2)	0.461
40–49, *n* (%)	68 (39.8)	43 (36.1)	25 (48.1)	0.142
50–59, *n* (%)	41 (24.0)	27 (22.7)	14 (26.9)	0.551
< 40 years, *n* (%)	62 (36.3)	49 (41.2)	13 (25.0)	0.043
≥ 40 years, *n* (%)	109 (63.7)	70 (58.8)	39 (75.0)	0.043
Gender, (male/female)	170/1	118/0	51/1	0.131
Body mass index, (kg/m^2^) (mean ± SD)	23.5 ± 3.2	23.5 ± 3.4	23.7 ± 2.7	0.636
Married, *n* (%)	160 (93.6)	108 (90.8)	51 (98.1)	0.119
Having children, *n* (%)	150 (87.7)	101 (84.9)	49 (94.2)	0.099
College education, *n* (%)	12 (7.0)	10 (8.4)	2 (3.8)	0.283
Occupation				
Mental worker, *n* (%)	41 (30.0)	39 (32.8)	2 (3.8)	0.002
Manual worker, *n* (%)	82 (48.0)	52 (43.7)	30 (57.7)	0.092
Farmer, *n* (%)	11 (6.4)	6 (5.0)	5 (9.6)	0.262
Self-employed, *n* (%)	29 (17.0)	18 (15.1)	11 (21.2)	0.334
Other, *n* (%)	8 (4.6)	4 (3.4)	4 (7.7)	0.217
Hometown region				
Central, *n* (%)	26 (15.2)	18 (15.1)	8 (15.4)	0.965
East, *n* (%)	124 (72.5)	89 (74.8)	35 (67.3)	0.313
West, *n* (%)	21 (12.3)	12 (10.1)	9 (17.3)	0.186
Cardiovascular risk factors				
Smoking history, *n* (%)	141 (82.5)	99 (83.2)	42 (80.8)	0.701
Hypertension, *n* (%)	12 (7.0)	8 (6.7)	4 (7.7)	0.819
Diabetes mellitus, *n* (%)	12 (7.0)	9 (7.6)	3 (5.8)	0.673
Hyperlipidaemia, *n* (%)	15 (8.8)	10 (8.4)	5 (9.6)	0.797
Cause of AICLI				
TAO, *n* (%)	159 (93.0)	112 (94.1)	47 (90.4)	0.379
Other, *n* (%)	12 (7.0)	7 (5.9)	5 (9.6)	0.379
Medication history				
Antiplatelet drugs, *n* (%)	104 (60.8)	77 (70.6)	27 (51.9)	0.115
Vasodilator, *n* (%)	39 (22.8)	19 (16.0)	13 (25.0)	0.164
Warfarin/Rivaroxaban, *n* (%)	18 (10.5)	11 (9.2)	7 (13.5)	0.408
Anti-infective treatment, *n* (%)	5 (2.9)	3 (2.5)	2 (3.8)	0.636
Surgical history				
Bypass, *n* (%)	6 (3.5)	4 (3.4)	2 (3.8)	0.874
Endarterectomy, *n* (%)	1 (0.6)	0 (0.0)	1 (1.9)	0.129
Stent grafting, *n* (%)	8 (4.7)	4 (3.4)	4 (7.7)	0.217
Balloon angioplasty, *n* (%)	25 (14.6)	18 (10.5)	7 (13.5)	0.777
Thrombolysis, *n* (%)	26 (15.2)	17 (9.9)	9 (17.3)	0.613
Thrombectomy, *n* (%)	12 (7.0)	8 (4.7)	4 (7.7)	0.819
Toe amputation, *n* (%)	19 (11.1)	13 (10.9)	6 (11.5)	0.906
Blood examination, *n* (%)				
Fibrinogen, mg/dL, (median, IQR)	321.5 (257–408)	307 (244–388)	354 (292–477)	< 0.001
CRP, mg/L, (median, IQR)	5.4 (1.8–14.8)	4.5 (1.55–11.65)	8.2 (2.3–18.7)	0.022
Glucose, mmol/L, (median, IQR)	4.7 (4.4–5.2)	4.7 (4.4–5.1)	4.8 (4.6–5.3)	0.915
GHb, %, (median, IQR)	5.4 (5.2–5.7)	5.45 (5.2–5.7)	5.4 (5.25–5.60)	0.359
Creatinine, μmol/L, (median, IQR)	71.5 (64–82)	72 (65–82)	71 (63–82)	0.600
GFR, mL/min, (median, IQR)	107 (99–119)	107 (97–117)	109 (101.5–120)	0.222
ESR, mm/h, (median, IQR)	14 (7–30)	14.5 (7–32)	13 (6–26)	0.453

The data presented are the numbers (%) and the means ± standard deviations or medians and the interquartile ranges

RTW, return to work; non-RTW, not return to work; SD, standard deviation; AICLI, angiitis-induced critical limb ischaemia; TAO, thromboangiitis obliterans; IQR, interquartile range; CRP, C-reactive protein; GHb, glycosylated haemoglobin; GFR, glomerular filtration rate; ESR, erythrocyte sedimentation rate

### RTW

**Fig. 2:**
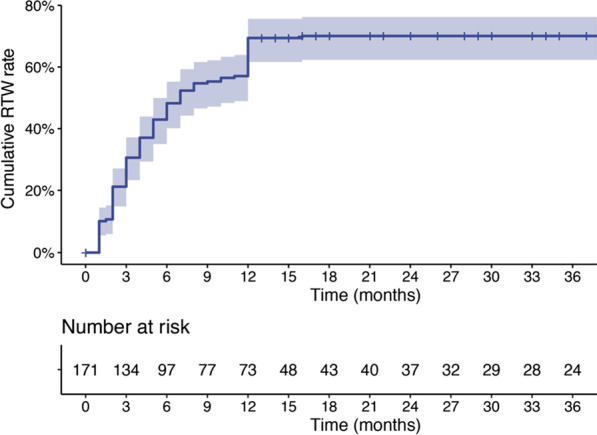
Kaplan–Meier curves showing the probabilities of cumulative RTW rate. RTW, return to work

All patients lost the ability to work before admission to the study. A total of 119 patients who returned to work within 12 months after cell transplantation were included in the RTW group, and the remaining 52 were included in the non-RTW group. The 12-month and 24-month RTW cumulative rates were 69.4% (95% confidence interval [CI] 61.6–75.6%) and 70.1% (95% CI 62.3–76.2%), respectively (Fig. 2). Among the 119 RTW patients, 82 patients (68.9%) returned to their preoperative jobs, 25 patients (21.0%) switched to easier jobs with fewer physical demands, and 12 patients (10.1%) changed their jobs for AICLI-unrelated reasons (Table 2). Fifty-two patients failed to return to work within 12 months after cell transplantation, among whom 19 patients (36.6%) were unable to work, 6 patients (11.5%) retired early, and 19 patients (36.5%) preferred not to work due to AICLI. The remaining eight patients (15.4%) were unable to or preferred not to work not directly owing to AICLI (1 for femoral fracture, 2 for anterior circulation cerebral infarction, 2 for COVID-19 epidemic, 1 for injury of common peroneal nerve and 2 for family affairs) (Table 2).

**Table 2:** Working status for AICLI patients at 12 months after cell transplantation

Working status	*n* = 171
RTW	
Return to the same work, *n* (%)	82 (48.0)
Change work owing to AICLI, *n* (%)	25 (14.6)
Change work owing to other reasons, *n* (%)	12 (7.0)
Non-RTW	
Unable to work owing to AICLI, *n* (%)	19 (11.1)
Early retirement, *n* (%)	6 (3.5)
Prefer not to work owing to AICLI, *n* (%)	19 (11.1)
Unable or preferred not to work not directly owing to AICLI, *n* (%)	8 (4.7)

AICLI, angiitis-induced critical limb ischaemia; RTW, return to work; non-RTW, not return to work

### Characteristics of RTW group and non-RTW group

The mean age of the RTW group was lower than that of the non-RTW group (40.9 ± 9.8 versus 44.3 ± 8.9 years, *P* = 0.033), and the frequency of patients aged < 40 years in the RTW group was significantly higher (41.2% versus 25.0%, *P* = 0.043) (Table 1). In addition, a significant difference was also observed in terms of the type of preoperative occupation between the two groups (Table 1). Although manual workers accounted for the largest proportion in both groups, the proportion of mental workers was significantly larger in the RTW group (32.8% vs 3.8%, *P* = 0.002). When the blood examinations were evaluated, patients in the RTW group had significantly lower CRP and blood fibrinogen levels than those in the non-RTW group (*P* = 0.022 and *P* < 0.001, respectively) (Table 1). No significant differences were observed between the RTW group and the non-RTW group in terms of other sociodemographic characteristics, aetiologies, risk factors for cardiovascular disease, or treatment histories (Table 1).

Regarding the ischaemic limbs, no significant differences were observed in terms of the number of ischaemic limbs, the RC, upper/lower limbs involved and test results such as ABI and TcPO_2_. Considering the highest level of arterial occlusion, five patients (2.9%) had an occlusion in the iliac artery (including the common and external iliac arteries), 71 (41.5%) in the femoral artery (including the common femoral artery and superficial femoral artery) or brachial artery, 26 (15.2%) in the popliteal artery and 69 (40.4%) in the arteries below the knee or elbow. Although no significant difference was observed between the two groups in terms of the highest level of arterial occlusion, the RTW group was characterized by a significantly higher percentage of patients in whom the highest arterial occlusion level was at the popliteal/below the knee or elbow arteries (60.5% versus 44.2%, *P* = 0.050) (Table 3). Although no significant difference was observed in terms of RC between the two groups (*P* = 0.401), among 150 patients with ulcer or gangrene (RC 5), 23 patients (13.4%) were also affected by perioperative infection, and a significantly lower percentage of patients with perioperative ulcer/gangrene infection was observed in the RTW group (8.4% vs 25.0%, *P* = 0.011) (Table 3). In terms of cell products, RTW patients were observed to have a significantly higher dosage of the total amount of CD34 + cells transplanted (43.8 × 10^6^ [25.4–97.2 × 10^6^] vs 29.8 × 10^6^ [15.3–52.9 × 10^6^], *P* = 0.002) and the amount of CD34 + cells transplanted per kg (6.8 × 10^5^/kg [3.6–12.0 × 10^5^/kg] vs 4.6 × 10^5^/kg [2.5–8.2 × 10^5^/kg], *P* = 0.012).

**Table 3:** Comparison of characteristics of ischaemic limbs and autoimplants between two groups of patients

	Total	RTW group	Non-RTW group	*P* value
	(*n* = 171)	(*n* = 119)	(*n* = 52)	
Number of ischaemic limbs				
1	136 (79.5)	93 (78.1)	43 (82.7)	0.498
2	30 (17.5)	22 (18.5)	8 (15.4)	0.625
3	3 (1.8)	2 (1.7)	1 (1.9)	0.912
4	2 (1.2)	2 (1.7)	0 (0.0)	0.347
Ulcer without gangrene, *n* (%)	67 (39.2)	49 (41.2)	18 (34.6)	0.419
Gangrene, *n* (%)	82 (48.0)	53 (44.5)	29 (55.8)	0.176
Upper limbs involved, *n* (%)	13 (7.6)	10 (9.7)	3 (5.8)	0.550
Lower limbs involved, *n* (%)	166 (97.1)	115 (96.6)	51 (98.1)	0.608
Highest level of arterial occlusion				
Iliac artery, *n* (%)	6 (3.5)	4 (3.3)	2 (3.8)	0.874
Femoral/brachial artery, *n* (%)	70 (40.9)	43 (36.1)	27 (51.9)	0.053
Popliteal artery, *n* (%)	26 (15.2)	22 (18.4)	4 (7.7)	0.065
Below the knee or elbow, *n* (%)	69 (40.4)	50 (42.0)	19 (36.5)	0.502
Iliac/femoral/brachial artery, *n* (%)	76 (44.4)	47 (39.5)	29 (55.8)	0.050
Popliteal/below the knee or elbow artery, *n* (%)	95 (55.6)	72 (60.5)	23 (44.2)	0.050
Rutherford class				
4, *n* (%)	22 (12.9)	17 (14.3)	5 (9.6)	0.401
5, *n* (%)	149 (87.1)	102 (85.7)	47 (90.4)	0.401
Ulcer/gangrene				
Ulcer/gangrene with infection	23 (13.4)	10 (8.4)	13 (25.0)	0.011
Ulcer/gangrene without infection	126 (73.7)	92 (77.3)	34 (65.4)	0.103
Without ulcer/gangrene	22 (12.9)	17 (14.3)	5 (9.6)	0.401
ABI^a^, (median, IQR)	0.49 (0.37–0.64)	0.48 (0.37–0.61)	0.53(0.35–0.81)	0.062
TcPO_2_, mmHg, (median, IQR)	19 (10–28)	18.5 (10–28)	19 (5–30)	0.496
Cell product				
Type				
PBMNCs, *n* (%)	97 (56.7)	69 (58.0)	28 (53.8)	0.615
PCCs, *n* (%)	74 (43.3)	50 (42.0)	24 (46.2)	
CD34^+^ cells, (10^6^), (median, IQR)	40.4 (23.4–79.1)	43.8 (25.4–97.2)	29.8 (15.3–52.9)	0.002
CD34^+^ cells/kg, (10^5^/Kg), (median, IQR)	5.8 (3.2–11.0)	6.8 (3.6–12.0)	4.6 (2.5–8.2)	0.012
Cell viability, %, (median, IQR)	98.4 (97.4–98.8)	98.4 (97.1–98.9)	98.2 (97.7–98.6)	0.826

The data presented are the numbers (%) and the means ± standard deviations or medians and the interquartile ranges

RTW, return to work; non-RTW, not return to work; IQR, interquartile range; ABI, ankle-brachial index; TcPO_2_, transcutaneous oxygen pressure; PBMNCs, peripheral blood mononuclear cells; PCCs, purified CD34^+^ cells

^a^ABIs of 158 patients with lower limbs treated were included in this analysis, while the other 13 patients with upper limbs treated were excluded

### Multivariate analysis

According to the results of the univariate logistic regression, several variables were screened out: age < 40 years (OR 2.107, 95% CI 1.034–4.293, *P* = 0.043), married (OR 0.193, 95% CI 0.024–1.532, *P* = 0.119), having children (OR 0.344, 95% CI 0.097–1.222, *P* = 0.099), highest arterial occlusion level at popliteal/below the knee or elbow arteries (OR 1.932, 95% CI 0.990–3.734, *P* = 0.050), perioperative limb infection of patients with ulcer or gangrene (OR 0.306, 95% CI 0.123–0.763, *P* = 0.011), preoperative occupation as mental workers (OR 7.962, 95% CI 2.334–27.159, *P* = 0.002), fibrinogen > 4 g/L (OR 0.453, 95% CI 0.218–0.939, *P* = 0.033), CRP > 3 mg/L (OR 0.589, 95% CI 0.284–1.223, *P* = 0.156), and base-10 logarithm (Log) of total amount of transplanted CD34+ cells (OR 2.824, 95% CI 1.337–5.961, *P* = 0.006) and Log of total amount of transplanted CD34+ cells per kg (OR 2.439, 95% CI 1.167–5.099, *P* = 0.018) (Table 4). After multivariate logistic analysis, age < 40 years (OR 2.659, 95% CI 1.138–6.719, *P* = 0.029) and preoperative job as mental workers (OR 8.930, 95% CI 2.665–42.847, *P* = 0.002) were identified as independent protective factors, and perioperative limb infection of patients with ulcer or gangrene (OR 0.250, 95% CI 0.075–0.779, *P* = 0.019) was identified as an independent risk factor for RTW (Table 4).

**Table 4:** Univariate and multivariate logistic regression analysis of independent risk factors

Candidate variable	Univariate analysis		Multivariate analysis	
	OR (95% CI)	*P* value	OR (95% CI)	*P* value
Age < 40 years	2.107 (1.034–4.293)	0.043	2.659 (1.138–6.719)	0.029
Married	0.193 (0.024–1.532)	0.119		
Having children	0.344 (0.097–1.222)	0.099		
Preoperative occupation as mental workers	7.962 (2.334–27.159)	0.002	8.930 (2.665–42.847)	0.002
CRP > 3 mg/L	0.589 (0.284–1.223)	0.156		
Fibrinogen > 4 g/L	0.453 (0.218–0.939)	0.033		
Highest arterial occlusion level at popliteal/below the knee artery	1.932 (0.999–3.734)	0.050		
Ulcer/gangrene with infection	0.306 (0.123–0.763)	0.011	0.250 (0.075–0.779)	0.019
Log (total transplanted CD34+ cell counts per kg)^a^	2.439 (1.167–5.099)	0.018		
Log (total transplanted CD34+ cell counts)^b^	2.824 (1.337–5.961)	0.006		

OR, odds ratio; CI, confidential interval; CRP, C-reaction protein

^a^Base-10 logarithm of total transplanted CD34^+^ cell counts per kg

^b^Base-10 logarithm of total transplanted CD34^+^ cell counts

## Discussion

Due to poor anatomical conditions and a high post-operative reocclusion rate, 20–50% of CLI patients, who are also referred to as no-option CLI (NO-CLI) patients, are not suitable for traditional interventions such as surgical or endovascular treatments [13]. AICLI, which shows a propensity for affecting the distal small vessels or microvessels and for destroying the anatomic runoff, represents a remarkable proportion of the NO-CLI population. However, AICLI patients, most of whom are TAO-induced, are characterized by a young age and are predominantly male. In the current study, 171 patients (170 were male) with a mean age of 41.9 ± 9.6 years were enrolled and analysed. Successful RTW is of vital significance not only to AICLI patients and their families but also to society. In this single-centre retrospective study, approximately two-thirds of the patients returned to work in the year after hospitalization for AICLI, indicating that cell therapy could not only result in the recovery of physical well-being but also contribute to satisfactory psychological health.

Vohra et al. reported that the post-operative employment status of patients with claudication who underwent lower limb revascularization was positively influenced by younger age [14]. Burger et al. reported that patients’ RTW after limb amputation depended on general factors, including age [15]. In the current study, age < 40 years old was an independent protective factor for AICLI patients’ RTW within 12 months. We infer that the role that age plays in RTW prognosis might be explained in several ways. First, the relief of ischaemia was partly due to the function of cell products, including angiogenesis and vasculogenesis. The autoimplants of older patients were reported to be related to impaired angiogenic potency, and younger age was reported to be associated with a better therapeutic effect of cell therapy in the treatment of NO-CLI patients [16–19]. Second, younger patients were characterized by better general health and fewer comorbidities, thus lowering the risk of adverse events, including death, organ dysfunction and cardio- and cerebrovascular diseases, which might occur during the follow-up and further stop patients from returning to work. In 2019, we reported a study in terms of factors associated with 6-month CLI remission among AICLI patients who underwent cell transplantation [20]. In a previous study, age ≥ 50 years old was reported to be an independent risk factor for 6-month CLI remission. However, in the current study, the age identified was younger (40 years old). We speculate that this may be partly explained by the fact that AICLI patients in their 50 s are more likely to retire early even after the total relief of CLI, given that the legal retirement age in China is 60 years old (for men) [21]. Although in studies of RTW in patients with coronary artery diseases or cerebral artery diseases, age was seldom reported to be a predictive factor [22–26], considering that cardio-cerebrovascular diseases tended to occur in the elderly population and most patients they enrolled in the studies were over 50 years old, it is reasonable that younger age was not reported to be associated with RTW.

Patients’ preoperative job type, together with education and income, are important indicators of socioeconomic status, and it may capture some or all of the effects of income and education [27]. Some studies also reported that different job types were indicators of patients’ RTW after orthopaedic surgery [28, 29]. In the current study, preoperatively working as a mental worker was identified as a positive independent risk factor for patients’ RTW. This can be reasonable because mental work generally demands less physical expenditure than other types of work, thus lowering the physical threshold needed for RTW. Among all the job types in the current study, mental workers had not only the highest RTW ratio (39/41, 95.1%) but also the highest return to the same work ratio (31/41, 75.6%). In contrast, for jobs that require a certain amount of physical activity, including manual workers and farmers, the proportions of non-RTW patients and patients who changed jobs due to AICLI were relatively high. As we mentioned before, job type could also reflect workers’ education to some extent. Jiang et al. reported that college education was associated with a higher likelihood of return to work after acute myocardial infarction [22]. Wang et al. reported that a higher educational level was a significant factor associated with RTW in patients with severe traumatic brain injury [30]. In the current study, 7.0% (12/171) of patients had a bachelor’s degree, and RTW was achieved in ten patients, of whom eight were preoperatively working as mental workers.

Out of 171 patients, 150 (87.7%) were in RC 5 with an ulcer or gangrene at admission, and 13.4% (23/171) of them had infections during the perioperative period. Infection is a factor that generally increases oxygen consumption and further aggravates the ischaemic degree and tissue loss in patients with CLI. Lu et al. reported that infection was an important independent risk factor for amputation in patients with diabetes-induced CLI [31]. On the other hand, patients with limb infection often require relatively long-term anti-infective treatment, including antibiotic treatment, regular dressing changes and even elective debridement. This process not only prolongs the time period for rehabilitation but also makes patients pay greater attention to their ischaemic limbs for fear of recurrence, thus delaying or even cancelling their plans to RTW.

RTW is associated with the relief of ischaemia after cell transplantation. However, not all patients return to work after total relief of CLI. Many RTW patients claimed to be free of pain in the transplanted limb and started working again within the 12-month follow-up, while a 2-class pain evaluated by the Wong–Baker Faces Pain Rating Scale (WBFPS) could still be observed. On the other hand, there were also many patients who were resistant to re-employment even after total relief of limb ischaemia, and one was even diagnosed with depression. Therefore, both physical and mental well-being are essential for RTW, and considering that the retirement age for many jobs in China has been increased to 65 years, RTW will be an increasingly important indicator in measuring treatment methods.

There are several limitations in the current study. The first and biggest limitation lies in its essence as a single-centre retrospective study, which could pose a risk of selection bias. The second limitation of the current study is the composite nature of RTW, which could be affected by many factors including cell therapy. The third limitation is the lack of a control group. Therefore, a prospective randomised controlled clinical trial is required to provide more definitive data in the future.

## Conclusion

AICLI patients who underwent cell transplantation had a satisfactory midterm RTW cumulative rate. AICLI patients < 40 years old with a preoperative occupation as a mental worker were more likely to return to work within 12 months after cell transplantation. For AICLI patients, the prevention of limb infection during the perioperative period is of great significance to RTW.

## Data Availability

The datasets generated during and/or analysed during the current study are available from the corresponding author on reasonable request. All authors have accessed the database and verified its accuracy.

## Abbreviations

RTW: Return to work
AICLI: Angiitis-induced critical limb ischaemia
PCCs: Purified CD34+ cells
PBMNCs: Peripheral blood mononuclear cells
